# Cachexia and Sarcopenia in Older Adults with Cancer: A Comprehensive Review

**DOI:** 10.3390/cancers11121861

**Published:** 2019-11-25

**Authors:** Richard F. Dunne, Kah Poh Loh, Grant R. Williams, Aminah Jatoi, Karen M. Mustian, Supriya G. Mohile

**Affiliations:** 1 Wilmot Cancer Institute, University of Rochester Medical Center, Rochester, NY 14642, USA; Kahpoh_Loh@urmc.rochester.edu (K.P.L.); karen_mustian@urmc.rochester.edu (K.M.M.); supriya_mohile@urmc.rochester.edu (S.G.M.); 2 University of Rochester NCI Community Oncology Research Program (UR NCORP), Rochester, NY 14642, USA; 3 Division of Hematology/Oncology, University of Alabama at Birmingham School of Medicine, Birmingham, AL 35233, USA; grwilliams@uabmc.edu; 4 Department of Oncology, Mayo Clinic, Rochester, MN 55905, USA; Jatoi.Aminah@mayo.edu

**Keywords:** cachexia, sarcopenia, geriatric oncology, geriatric assessment, weight loss, muscle, wasting

## Abstract

Cancer cachexia is a syndrome characterized by weight loss with accompanying loss of muscle and/or fat mass and leads to impaired patient function and physical performance and is associated with a poor prognosis. It is prevalent in older adults with cancer; age-associated physiologic muscle wasting and weakness, also known as sarcopenia, can compound deficits associated with cancer cachexia in older adults and makes studying this condition more complex in this population. Multiple measurement options are available to assess the older patient with cancer and cachexia and/or sarcopenia including anthropometric measures, imaging modalities such as Dual X-ray absorptiometry (DEXA) and Computed Tomography (CT), muscular strength and physical performance testing, and patient-reported outcomes (PROs). A geriatric assessment (GA) is a useful tool when studying the older patient with cachexia given its comprehensive ability to capture aging-sensitive PROs. Interventions focused on nutrition and increasing physical activity may improve outcomes in older adults with cachexia. Efforts to develop targeted pharmacologic therapies with cachexia have not been successful thus far. Formal treatment guidelines, an updated consensus definition for cancer cachexia and the development of a widely adapted assessment tool, much like the GA utilized in geriatric oncology, could help advance the field of cancer cachexia over the next decade.

## 1. Introduction

Cancer cachexia is a prevalent and debilitating syndrome characterized by weight loss with concomitant loss of muscle and/or fat mass [1]. Cancer cachexia leads to functional impairment, reduced physical performance and poorer survival [1]. Cachexia is driven by a negative energy balance that can result from anorexia, which is influenced hormonally through a network of neuropeptides and mechanically by tumor, as well as by a pro-inflammatory environment that generates hypermetabolism [2,3]. Cachexia can develop in the setting of a wide range of tumor types, but it is most common in cancers of the upper gastrointestinal tract (GI) and lung, occurring in upwards of 83% of patients with pancreatic and gastric cancers and 60% of those with lung cancer [4]. Despite decades of research, no United States Food and Drug Administration (FDA) approved standard treatment exists; therapeutic development in the field of cancer cachexia remains a critical unmet need.

Unfortunately, the incidence of cancer cachexia is likely to grow in years to come. The United States population is aging, and the incidence of cancer diagnoses in adults over the age of 65 is expected to increase by 67% by 2030 when compared to 2010 [5]. Furthermore, cancers with the highest rates of associated cachexia occur predominantly in older adults. Compounding the problem is that physiologic age-related loss of muscle mass and muscle function could occur as well, a process historically referred to as sarcopenia [6]. These factors create a significant challenge for healthcare providers caring for older adults with cancer over the years to come.

The term cachexia is commonly interchanged with sarcopenia, though these represent separate entities with significant overlap. We describe the prevalence of cachexia and sarcopenia in older adults with cancer here and provide an in-depth discussion of terminology and clinical definitions below. Cancer cachexia, diagnosed by an examination of weight loss, body mass index (BMI) and skeletal muscle, is very common in older adults with cancer. Among patients referred to a specialized geriatric oncology clinic to undergo a geriatric assessment, up to 65% were found to have cancer cachexia [7]. Sarcopenia, which is defined commonly as a combination of low muscle strength, reduced muscle mass, and/or reduced physical performance, can be found in anywhere from 12.5% to 57.7% of the geriatric cancer population [8,9,10,11]. 

The geriatric assessment (GA) evaluates physical, functional, social, and psychological well-being and includes an assessment of weight loss and nutrition [12]. The GA possesses great potential as a tool with which to study cachexia and wasting disorders in older adults [13]. Nevertheless, cachexia remains understudied in this population, and older adults continue to be poorly represented in randomized phase II and III cancer cachexia clinical trials [14,15,16,17,18]. Cachexia trials designed specifically for older adults with cancer or with broader inclusion criteria to allow inclusion of older adults are urgently needed. While awaiting future trial design, subgroup analyses of older adults in published cachexia trials or the collection of real-world retrospective data from the oncology clinic could enhance our understanding of cachexia in this population.

In this review, we will analyze commonly used definitions for cachexia and sarcopenia in the context of older adults with cancer, discuss how cachexia and muscle deficiencies are measured, describe outcomes associated with these disorders in older adults, and explore interventions for older adults with cachexia and sarcopenia.

## 2. Cachexia and Sarcopenia in Older Adults with Cancer: One and the Same?

The hallmarks of both cachexia and sarcopenia center around muscle loss, allowing for significant overlap between the two conditions. Clinical definitions of the two, however, have evolved in recent years to focus on separate relevant metrics. Discussed in depth below, cachexia is most commonly defined clinically by weight, muscle, and fat tissue loss [1], whereas the most recent consensus definition of sarcopenia relies primarily on muscle function (Table 1) [8].

Earlier definitions of cachexia focused on weight, physical performance, and patient function and have varied over time. The Cachexia Consensus Conference in 2006 published diagnostic criteria that defined cachexia as 5% weight loss in the previous 6 months along with three of the following: reduced muscle strength, fatigue, anorexia, reduced fat-free mass, or systemic signs of inflammation [19]. In 2009, the Screening the Nutritional Status in Oncology (SCRINO) working group defined cachexia as greater than 10% weight loss and established classes of cachexia based on anorexia, early satiety, and fatigue [20]. In 2011, an international consensus definition and classification of cancer cachexia was published, defining cancer cachexia as greater than 5% weight loss in the previous 6 months or 2%–5% weight loss with either a BMI of <20 kg/m^2^ or reduced muscle mass [1]. This definition is considered by many as the gold standard of cancer cachexia and has been validated in a follow-up study of 861 subjects (mean age: 62 years) with and without cachexia [21]. Relying solely on weight and quantitative muscle mass to clinically characterize older adults, however, could be problematic. In this population, muscle mass and muscle function may not change in conjunction with one another nor will deficits in mass and function necessarily occur in a linear fashion [22,23,24]. This was illustrated in a study of 734 patients with lung cancer where patient function and quality of life did not significantly change until muscle mass dropped below a certain threshold value [25]. Therefore, the use of quantitative muscle mass alone may over- or under-estimate the prevalence of wasting disorders and may make patient selection for therapies more challenging. A more comprehensive evaluation that incorporates performance and physical function may be more useful in the geriatric oncology population.

In recent body composition-related oncology research, many studies use the term “sarcopenia” to refer to a quantitative reduction in muscle mass without qualifiers. However, this may not be an appropriate use of this term [24]. Sarcopenia was originally described as a physiologic age-related loss of muscle [26], and has most recently been defined by experts to indicate both a loss of muscle and muscle function. In 2010, the European Working Group on Sarcopenia in Older People (EWGSOP) developed a consensus definition of sarcopenia that required low muscle mass with one of the following: low muscle strength or reduced physical performance [27]. In 2019, EWGSOP published a revised consensus definition of sarcopenia based on updated research. Sarcopenia is now described as “a progressive and generalized skeletal muscle disorder that is associated with increased likelihood of adverse outcomes including falls, fractures, physical disability and mortality” [8]. EWGSOP notes in its updated report that diminished muscle strength is more closely tied to poor outcomes than reduced muscle mass. This finding is reflected in new diagnostic criteria for sarcopenia, which have now shifted to an emphasis on muscle strength rather than mass. The updated criteria specify three important points: (1) Low muscle strength indicates the diagnosis of sarcopenia is probable, (2) the diagnosis of sarcopenia is confirmed in the presence of low muscle strength and low muscle quantity or quality and (3) sarcopenia is considered severe if reduced physical performance is demonstrated along with reduced muscle strength and mass [8]. Although many patients with sarcopenia also have weight loss, change in weight is not integrated into the definition of sarcopenia.

In summary, the terms cachexia and sarcopenia describe debilitating disorders of muscle deficiency that reduce patient function and physical performance. Despite their overlap, they describe two distinct entities and the terms are not interchangeable. Sarcopenia is a muscle disorder best characterized by decreased strength and can be caused by a multitude of factors including normal aging and physical inactivity or can occur secondary to disease. Cachexia is a syndrome of muscle and/or fat wasting caused by disease (e.g., cancer, chronic obstructive pulmonary disease (COPD), HIV/AIDS) that is most widely characterized clinically by weight loss. In other words, many patients with cachexia have sarcopenia, meaning they manifest loss of muscle and muscle strength as part of their condition. Patients with sarcopenia, though, may not fit the criteria for cachexia because they lack associated weight loss or because changes in their muscle are not due to disease, but are physiologic. 

Older patients with cancer characterized as suffering from cachexia or sarcopenia phenotypically resemble one another and each condition can result in similar complications. It remains unclear at this time which term is most useful in describing wasting disorders in this population. With this in mind, we investigate tools, research studies, and treatment strategies applicable to both conditions. 

## 3. Measurement of Cachexia and Sarcopenia in Older Adults

Various instruments are used to assess cachexia and sarcopenia, and they can be broadly divided into objective and patient-reported measures (PROs). Table 2 provides a summary of objective tools to measure muscle mass directly or indirectly, muscle strength and muscle performance. Two of the most common yet simplest measures used in cachexia are weight loss and BMI [19]. These measures may be integrated into more comprehensive nutritional assessments such as the Mini-Nutritional Assessment (MNA) [28,29] and the Malnutrition Universal Screening Tool [30]. Beyond weight and body mass index, the MNA also assesses diet via questionnaire, includes a subjective assessment of patient health and nutrition, and globally assesses lifestyle, mobility, and medications. MNA has been validated in the geriatric population and is commonly used in studies of older adults with cancer [31]. 

For objective body composition (muscle, fat, lean mass) assessment, anthropometric measurement (e.g., BMI, calf circumference, and skinfold thickness) is the least expensive method, but there is a loss of accuracy with advancing age [38,39]. Several modalities with higher validity have recently been adapted to measure skeletal muscle mass, such as dual energy X-ray absorptiometry (DEXA), bioelectrical impedance analysis (BIA), computed tomography (CT) scan, and magnetic resonance imaging (MRI) [40]. Cross-sectional imaging (CT and MRI) can accurately assess total skeletal muscle mass by measuring muscle area on a single image slice, most commonly at the L3 vertebra, and, after accounting for stature, is reported as the skeletal muscle index. Assessment of body composition by cross-sectional imaging is the preferred muscle mass measurement in cancer cachexia over DEXA according to the international consensus panel on cachexia [1,41]. CT and MRI scans also have the advantage of measuring both muscle cross-sectional area and muscle quality by assessing muscle radiodensity. Muscle density, as measured by Hounsfield units on CT, is inversely related to muscle lipid content and is a surrogate for muscle quality [42]. 

Despite multiple benefits, however, none of these imaging modalities measure muscle strength and/or physical performance, which are important components of sarcopenia. To that end, several objective instruments have been developed. These measures are commonly incorporated as part of the GA described earlier. Some examples include handgrip strength, which was endorsed by the EWGSOP and the international consensus panel on cancer cachexia and has been validated in older adults [1,27], 6-minute walk test, gait speed test, and Short Physical Performance Battery (SPPB) [43]. A limitation is that definitions for impaired physical performance and functional status vary among and within these different measures. 

Among patient-reported measures used to assess sarcopenia, a 14-item questionnaire for sarcopenia (SarcoPRO) is associated with limitations in instrumental activities of daily living (IADLS) in older adults with cancer [36]. This tool was developed by conducting open-ended interviews to understand the effects of reduced muscle strength on function in older adults with known sarcopenia [44]. SarcoPRO also correlated moderately with the short physical performance battery, a validated measure associated with morbidity and mortality in older adults [36,45,46]. SarcoPRO is useful in settings where imaging is not routinely or easily obtained. SARC-F is another 5-item patient-reported instrument [47]. While its sensitivity is low in older adults, its high specificity may be utilized to select patients for further assessment of sarcopenia.

Though an array of assessment instruments is available to clinicians, formal cachexia and sarcopenia measurements, except for weight and/or BMI, are not commonly integrated into clinical practice. Vulnerabilities associated with muscle loss such as functional impairment or reduced physical performance are often not captured in routine oncology visits; current guidelines seek to improve this. One fundamental strategy is the use of a GA in the oncology clinic, which is recommended for all older adults with cancer by the American Society of Clinical Oncology (ASCO), the National Comprehensive Cancer Network, and the International Society of Geriatric Oncology [43,48,49,50]. Despite these recommendations, the implementation of the GA in daily oncology practice has been slow; this is, in part, due to the perception that it is time consuming [51]. However, the ASCO guideline notes that the GA is feasible to complete in both clinical trials and in the oncology clinic, takes about 20–30 minutes to complete, and can be obtained via a paper or electronic record [43].

We argue that the GA is crucial in the evaluation of the older patient with cancer cachexia. The assessment of the patient with cachexia recommended by the international consensus panel on cancer cachexia strongly resembles the geriatric assessment (Table 3 and Table 4). This recommendation includes evaluation of anorexia, hypermetabolism/inflammation, muscle mass and strength, physical performance, and psychosocial impairment (Table 3). For comparison, the ASCO Geriatric Oncology Guidelines recommend that the following data be captured by the GA: chemotherapy toxicity risk assessment, life expectancy estimate, functional assessment (i.e., IADLs), comorbidity evaluation, screening for falls, screening for depression, assessment of cognition, and screening for malnutrition (e.g., weight loss, MNA) [48]. The majority of the assessment is patient-reported and can be completed by patient/caregiver with minimal assistance in the waiting or exam room [43]. The guidelines also recommend, if possible, completing objective measures of physical performance like the SPPB, Timed Up and Go (TUG), and gait speed (Table 4). These performance tests do not require special equipment and can be carried out by a nurse, research assistant or medical technician. The creation of formal guidelines and increasing utilization of the GA creates an opportunity to screen and diagnose cachexia and sarcopenia in older adults with cancer and can bring further awareness to these conditions.

## 4. Outcomes in Older Adults with Cancer and Wasting Disorders

The association of cancer cachexia with adverse outcomes and poor prognosis is well-documented [1,21,52]. Data are limited, however, regarding cachexia specifically in older adults with cancer. A systematic review published in 2017 sought studies evaluating how nutritional deficiencies and/or cachexia affects chemotherapy administration and outcomes in the geriatric oncology population. Multiple studies were identified examining malnutrition in this population, principally using the MNA tool, but no studies examining cachexia specifically in older adults were found [53]. The systematic review found lower MNA scores at diagnosis (score 0–14, 0–7 malnourished, 8–11 at risk for malnutrition) to be associated with increased toxicity from chemotherapy and a worse prognosis [53]. 

More recently, a single-center retrospective analysis examined cachexia and its association with the GA and survival in 100 older adults with solid tumors [7]. All patients underwent a GA; the mean age was 80 years. Using the international consensus definition, cachexia was defined in this study as 5% weight loss or 2–5% weight loss with accompanying muscle depletion as measured by CT scan. Cachexia was found to be associated with functional impairment (*p* = 0.017) and worse survival (1 year in patients with cachexia vs. 2.1 years in patients without cachexia; *p* = 0.011). In the same study, neither weight loss nor muscle loss individually was associated with survival or functional impairment [7].

Underlying the paucity of cachexia research in older adults and in general is the difficulty of obtaining accurate data on weight loss and nutrition retrospectively. There is an abundance of data, however, on archived CT scans readily available in oncology clinics globally, which allow retrospective evaluation of quantitative muscle mass, muscle quality, and fat mass. This tactic has spawned a host of studies in all age groups that have demonstrated correlations between skeletal muscle depletion and various outcomes, including increased complications from surgery [54,55], toxicity from chemotherapy [56,57], and, most notably, poorer overall survival [58,59,60]. In older adults with cancer, however, correlations between skeletal muscle depletion alone and adverse outcomes are less clear.

A report of 341 patients undergoing esophagectomy for esophageal cancer, stratified by age (≥ or <65 years), investigated associations between skeletal muscle quantity as measured by CT skeletal muscle index and surgical outcomes and survival. In the older cohort (166 patients), compared to patients with adequate muscle mass, skeletal muscle depletion was associated with worse in-hospital mortality (6.8% vs. 0.0%, *p* = 0.037), higher rates of anastomotic leaks (31.5% vs. 15.2%, *p* = 0.015), and poorer 5-year overall survival rates (26.5% vs. 56%, *p* < 0.0001) [61]. Additionally, in a study of 70 older patients (median age: 71 years) who received neoadjuvant or adjuvant chemoradiation for rectal cancer, low skeletal muscle index (muscle depletion) was associated with a higher hazard ratio (HR) for death (6.01; *p* = 0.001). This HR was higher than that for age, sex, cancer stage and carcinoembryonic antigen (CEA) level [62].

Several studies, however, have found that alternative measures used to characterize muscle were more informative than quantitative muscle mass indices in older adults with cancer. Examining muscle quality in addition to muscle quantity has garnered increasing interest of late. Muscle quality is particularly relevant in the geriatric oncology population. In a study of 734 patients with lung cancer of all ages (mean age: 65 years) referenced earlier, men and women aged greater than 75 years of age had significantly reduced muscle quality compared to those less than 75 years old [25]. In a report of 162 older adults with cancer from the Carolina Senior Registry (median age: 71 years), muscle quantity assessed via CT scan was not associated with a standard frailty index [63]. On the other hand, muscle density (quality) was more closely associated with frailty in this population. A separate published report examining 185 older adults with cancer from the same Carolina Senior Registry (mean age: 73 years) also demonstrated that muscle density, and not muscle mass, was associated with functional impairment as measured by IADLs, walking, stair-climbing, and the Timed Up and Go test [24]. A separate study also demonstrated lower muscle quality was associated with major post-operative complications including anastomotic leaks, intensive care unit admissions, longer hospital stays, and higher readmission rates in 373 older adults (median age: 78 years) undergoing surgery for colorectal cancer [64].

Reduced muscle strength and physical performance are important factors in identifying sarcopenia and also have been tied to adverse outcomes in the geriatric oncology population. In a study of 103 older adults with advanced cancer (mean age: 70 years), muscle strength, and not muscle mass or muscle density, was significantly associated with overall survival [65]. A separate study of 197 older adults (mean age: 76 years) undergoing abdominal cancer surgery had similar findings. Improved physical performance as measured by 6-minute walking test distance, gait speed, handgrip strength, and self-reported physical activity was predictive of the likelihood of patients being discharged to home rather than to a nursing facility [66].

Though adipose tissue wasting is not incorporated into standard diagnostic criteria of cachexia or sarcopenia, it remains an important characteristic in patients suffering from cancer-associated cachexia. In animal studies, adipose tissue breakdown has been described as an essential component in the pathophysiology of cancer cachexia and that blocking lipolysis may be an important treatment strategy [67]. In humans, fat mass can also be accurately ascertained through cross-sectional imaging [68] and has been associated with worse survival in patients with advanced cancer [69]. This has been corroborated in older adults with cancer as well. In a study of 80 adults older than 70 years of age with Diffuse Large B-Cell Lymphoma, fat wasting was associated with reduced progression free survival and overall survival (*p* = 0.0042 and 0.0342, respectively) [70]. 

In summary, it is evident that predicting adverse outcomes in older adults with cancer using measures of body composition can be quite complex. Sarcopenia can be a normal physiologic process of aging that can be further compounded by additional weight and muscle loss resulting from metabolic and inflammatory changes induced by cancer. A single measure such as weight, muscle index (muscle mass) or muscle density (muscle quality) is likely insufficient to fully characterize or predict outcomes in older adults with wasting disorders and cancer. Prospective studies that can comprehensively capture serial measures of physical performance, patient function, and patient-reported outcomes (including weight loss) and utilize objective, accurate and practical measures of muscle mass are sorely needed. 

## 5. Interventions for Cachexia and Sarcopenia in Older Adults

Strategies to efficaciously treat both cachexia and sarcopenia have similar intentions: (1) improve muscle mass, (2) improve muscle function and overall patient function, (3) and improve physical performance. As such, many of the interventions that we explore can likely apply to both conditions; clinical investigation of these strategies, however, usually focuses on populations with either cachexia, sarcopenia, or surrogate conditions like muscle wasting or weight loss. Optimal therapeutic advances will ultimately hinge on precisely targeting the underlying mechanisms of these conditions and could potentially lead to a divergence of strategies for treating cachexia and sarcopenia in the future.

While there is no FDA approved treatment for cancer cachexia, several interventions tailored to the individual are available to combat cachexia and sarcopenia. Given the multidimensional nature of these conditions, multimodal interventions are often associated with the best outcomes [71]. Adequate nutrition and resistance exercise are cornerstones of the management of sarcopenia [72] and are instrumental components of treatment approaches recommended by cachexia experts [71]. Although the evidence is not conclusive and no universal guidelines exist, the maintenance or generation of muscle mass requires adequate caloric and protein intake [73,74]. In addition to ensuring adequate caloric intake, protein supplementation and optimization of vitamin D levels are the most promising dietary strategies for age-related sarcopenia in non-oncologic studies [73]. For a nutritional intervention to be effective, it should provide sufficient calories, contain appropriate nutrients, and be of sufficient duration to affect muscle health [74]. Treating cachexia and sarcopenia effectively, however, entails a more sophisticated approach then merely eating more. Adequate caloric intake and nutritional supplementation alone are frequently unsuccessful in reversing or restoring muscle mass in patients suffering from cachexia [75]. Appetite stimulants (e.g., megestrol, steroids, and cannabinoids), which have been studied in patients with cachexia for decades, have aided in weight gain but have failed to improve other salient outcomes like physical performance and survival [76,77,78]. Furthermore, older adults with cancer may be at higher risk from toxicities associated with agents like corticosteroids and megestrol. Close examination of the risks and benefits of appetite stimulants is warranted in this population. 

Resistance training and aerobic training have been shown to increase muscle strength and function and represent an attractive treatment strategy for cachexia and sarcopenia [79]. No pharmacologic or nutritional intervention in the field to date has shown results superior to exercise [73]. Exercise and physical activity can reduce inflammation [80], induce molecular signaling pathways that support building muscle mass, and stimulate beneficial metabolic adaptations [81]. Two separate randomized studies in men with prostate cancer receiving androgen deprivation therapy (ADT), a population in which muscle loss is common, demonstrated that exercise may help those with cachexia and/or sarcopenia. In a study of 121 men with prostate cancer on ADT, the cohort of men older than 65 years randomized to resistance exercise demonstrated preserved lean mass, whereas those who were randomized to aerobic exercise or usual care demonstrated loss of lean mass [82]. In a study of a similar population, a combined resistance and aerobic exercise program helped reverse muscle loss [83]. Recently, researchers evaluated the effects of exercise on muscle mass in patients with early stage breast cancer receiving adjuvant chemotherapy. They randomized patients to resistance exercise training (*n* = 64), aerobic exercise training (*n* = 66), or usual care (*n* = 70) and demonstrated that resistance exercise can reverse sarcopenia and improve quality of life [84]. Multiple other studies in older adults with cancer at risk for cachexia and/or sarcopenia have also demonstrated that exercise can improve muscular strength and physical performance [85,86,87]. Based on the results of these studies and many others that examined the benefits of exercise on physical functioning and quality of life, the American College of Sports Medicine (ACSM) has developed exercise guidelines for adults with cancer [88]. Nonetheless, major barriers exist to implementing this simple intervention, as many community-dwelling adults lack access or motivation to partake in a rigorous exercise program [89] and little data or guidance is available specific to older adults with cancer. 

Clinical trials investigating multimodal interventions including exercise and nutrition targeting cachexia in older adults with cancer are needed. Evaluation of an early multimodal intervention consisting of both an exercise intervention and nutrition sessions in older adults with advanced pancreatic cancer and non-small cell lung cancer has demonstrated feasibility and is currently accruing as a randomized phase II study [90]. Also, a recently completed randomized controlled trial of a cancer rehabilitation program in older adults with cancer showed promise for increasing activity expectations and self-efficacy, although it did not improve functional status [91]. 

In addition to physical activity and nutrition, ideal multimodal treatment approaches include targeting mechanisms of cachexia. Inflammation, which can also be targeted by exercise and nutritional supplements, is a key underlying pathophysiologic mechanism of cachexia. Pharmacologic agents targeting inflammatory cytokines have been explored, though results are mixed [73]. Studies have previously investigated non-steroidal anti-inflammatory (NSAID) agents, anti-tumor necrosis factor (TNF) agents, thalidomide, and omega fatty acids with variable results [92]. Although several studies suggested a potential benefit of NSAID treatments in improving muscle mass and body weight in cancer cachexia, most studies have been small, methodologically flawed, and lacking a comparator [93]. Currently in progress is the promising MENAC trial, which is investigating a multimodal intervention that includes exercise, nutrition and ibuprofen in adults with cancer cachexia [NCT 02330926, EudraCT 2013-002282-19]. This study could have broad implications for the treatment of cancer cachexia and the design of future intervention trials.

Targeting hormonal pathways that exert influence on human metabolism and muscle have shown promise in early phase cachexia studies but we have yet to see positive phase III results. For example, selective androgen receptor modulators (SARMs) selectively bind to the androgen receptor and have been developed and tested in Phase I, II, and III trials as treatments for muscle wasting. Studies of enobosarm have shown increases in muscle mass and physical function [94]. Unfortunately, two Phase III studies investigating enobosarm, POWER I and POWER II, were negative, and this agent is no longer being investigated for the treatment of cachexia. An alternative endocrine pathway, activation of the ghrelin receptor with ghrelin agonists, has garnered significant interest for the treatment of cachexia over the past decade. Ghrelin is considered a “meal-initiating” hormone that is released by the stomach in response to prolonged fasting. Ghrelin agonists have demonstrated significant increases in food intake, body weight, and lean mass [73,95,96]. In a recent report of two large phase III trials (ROMANA I and ROMANA II) investigating the oral ghrelin receptor agonist anamorelin, significant increases in lean body mass and anorexia and cachexia symptoms over a 12-week period were demonstrated. These gains, unfortunately, were not accompanied by increases in handgrip strength, the co-primary endpoint, and, ultimately, did not result in FDA approval [14,97]. Although these agents improved lean mass, neither improved the physical performance or functional benchmarks set to ultimately measure efficacy [98]. Despite “negative” findings of the ROMANA trials, anamorelin remains in development for the treatment of cancer cachexia. There are currently two ongoing clinical trials [NCT 03743064 and 03743051] investigating the use of anamorelin to treat cancer-associated weight loss in patients with non-small cell lung cancer. The primary outcome of these studies is a “Composite Clinical Response,” which includes changes in weight and a patient-reported anorexia scale.

Treatment strategies for both cachexia and sarcopenia in the older adult should include recommendations for physical activity and guidance on adequate caloric and protein intake and dietary supplementation that may help achieve these goals. Appetite stimulants have had limited efficacy in cachexia clinical trials and can be associated with adverse effects, especially in older adults; short courses could be helpful in select patients. How best to utilize combinations of these interventions to improve outcomes in older adults with cancer is unclear at this time. More rigorously designed studies investigating multimodal treatment strategies of diet, physical activity, and pharmacologic agents targeting cachexia are needed. In addition to well-designed interventions, more focus and agreement on the proper endpoints for these intervention studies are necessary in order to move the field forward [99]. 

## 6. Future Directions

The development of a consensus definition of cancer cachexia [1] in 2011 and its validation [21] represent significant advances in this field. Standard diagnostic criteria allow researchers to identify subjects with cancer cachexia to study and provide more guidance for inclusion criteria in both prospective observation and clinical intervention trials. Nevertheless, wide recognition and implementation of the international consensus definition are lagging behind. The cause is currently unclear, but it may be related to overlapping definitions and confusing terminology that were discussed earlier. Furthermore, the lack of consensus guidelines for the treatment of cancer cachexia and the absence of a unified direction of research priorities in this field may also contribute. ASCO is currently developing guidelines for cachexia, a task that will hopefully enhance our integration and implementation of cancer cachexia care in clinical trials and daily oncology practice. In addition to formal guidelines, an updated consensus definition of cancer cachexia would likely help solidify terminology, awareness, diagnostic criteria and research priorities moving forward.

The identification and care of the older patient with cancer cachexia are challenging. ASCO Geriatric Oncology Guidelines recommend that a GA be performed in patients older than 65 receiving chemotherapy “to identify vulnerabilities that are not routinely captured in oncology assessments [43].” By performing a GA, impairments in weight, nutrition, patient function, physical performance, and psychosocial domains are readily identified, all of which are crucial to the evaluation of cancer cachexia. 

In addition to providing a consensus definition and classification of cancer cachexia, the cachexia research community must implement a standardized assessment, much like the geriatric oncology community has implemented the GA. Undoubtedly, a large effort is required; however, a “comprehensive cachexia assessment” could take the field to new heights. Assessment tools for cachexia have been developed previously such as the CASCO score, which utilizes measurement of medical history, physical performance, blood biomarkers and PROs [100], and the Functional Assessment of Anorexia and Cachexia Therapy (FAACT) scale [101], a validated PRO. These measurements, unfortunately, have not been widely adapted and typically have been used only for research purposes. A universal assessment that could be employed both in oncology clinics and clinical trials would launch new opportunities to study the syndrome of cachexia and, ultimately, help tailor multi-modal interventions to treat this complex problem.

## 7. Conclusions

Normal aging can result in sarcopenia, a condition defined by reduced muscular strength, depletion of muscle mass and/or reduced physical performance. As such, older patients with cancer are at significant risk for cancer cachexia, a syndrome defined by loss of weight and muscle mass and/or low BMI that is associated with increased toxicity from chemotherapy and a poor prognosis. The interplay between physiologic sarcopenia and cancer cachexia is, in part, responsible for the complexity that exists in studying wasting disorders in the geriatric oncology population. Comprehensive assessment with both patient-reported and objective measures of weight, nutrition, muscle mass, muscle quality, physical function, and physical performance is crucial in the evaluation of the older patient with cancer cachexia. Interventions for older adults with cancer cachexia should focus on improving nutrition and increasing physical activity, while pharmacologic treatments remain in development. Utilization of the GA in this population could be adapted to better study cachexia in older adults and may help improve the treatment of individuals with cachexia in daily oncology practice. 

## Figures and Tables

**Table 1:** The diagnostic criteria of cancer cachexia based on an international consensus definition and classification [1] as compared to European Working Group on Sarcopenia in Older People definition of sarcopenia [9].

Cancer Cachexia	Sarcopenia
>5% weight loss in the previous 6 months Or >2% weight loss and one of the following: (1) Body mass index < 20 kg/m^2^ (2) Evidence of muscle depletion. Example provided-appendicular skeletal muscle index consistent with sarcopenia (<7.26 kg/m^2^ in males and <5.45 kg/m^2^ in females)	(1) Diagnosis of Sarcopenia is probable with low muscle strength (2) Diagnosis is confirmed with low muscle quantity or quality. (3) Reduced physical performance along with reduced muscle strength and muscle quality/quantity represents severe sarcopenia.

**Table 2:** Tools to objectively measure impairments in muscle [32,33,34,35,36,37].

Tool	Advantages	Disadvantages
Weight	Practical, cheap, completed at each oncology visit	Prone to inaccuracies as does not take into account changes in fat mass and non-skeletal muscle
Muscle Mass		
CT or MRI [32]	Gold standard, can accurately assess muscle and fat mass, can detail individual muscle/muscle groups, are obtained as SOC in oncology patients and can be performed serially	Data collection and interpretation requires software and expertise and can be time consuming. Automated systems in development.
DEXA [32]	Cheap, minor radiation exposure, accurate measure of muscle mass	Not used in routine oncology practice, does not provide information on specific muscle/muscle groups
BIA [32]	Portable, uses electric current, no radiation exposure	Not used in routine oncology practice, less accurate, skewed by edema or use of diuretics
Muscle Quality (Muscle Density) [33]	CT can measure muscle density accurately by Hounsfield units, low variance, reliable	Indirect data of fat content within muscle, which is used to determine muscle quality
Muscle Strength		
Isokenetic muscle strength testing [34]	Gold standard for muscle strength testing, can provide force, endurance, torque, power	Requires expensive equipment that is not portable, nor widely available
Handgrip Dynamometry [35]	Cheaper than isokinetic testing and is portable, valid and reliable	Multiple protocols used, cross-study comparison difficult, may not be most representative of patient function
Physical Performance		
SPPB [36]	Composite score of tandem walk (balance), chair stands (functional testing) and gait speed), validated in older adults	Requires trained staff to conduct
6-minute walk test (measures speed or VO2 max) [37]	Excellent measure of functional capacity as measures submaximal cardiorespiratory fitness, cheap, easy to conduct	May be difficult for older patients with cachexia, healthcare provider supervision may be needed

Abbreviations. CT: computed tomography, MRI: magnetic resonance imaging, DEXA: dual energy X-ray absorpimetry, BIA: bioelectrical impedance, SPPB: Short Physical Performance Battery, VO2 max: maximal aerobic capacity.

**Table 3:** Recommended assessment of the cachectic patient as stated in the international consensus definition of cancer cachexia [1].

Characterization of the Cachectic Patient	Tools/Measures
Anorexia/Food Intake	Patient-reported protein/calorie intake, assessment of appetite
Hypermetabolism/Inflammation	No clear consensus but CRP noted as most widely used.
Muscle Mass and Strength	No clear consensus, panel preferred in order: cross-sectional imaging (CT or MRI), DEXA, anthropometry, and BIA. Hand-grip preferred over lower-limb extension for strength.
Function	Patient-reported function as per EORTC QLQ-C30 or physician-reported Karnofsky Score
Psychosocial	Assessment of distress about eating and weight

Abbreviations. CRP: C-reactive protein, CT: computed tomography, MRI: magnetic resonance imaging, DEXA: dual energy X-ray absorptiometry, BIA: bioelectrical impedance, EORTC QLQ-C30: European Organisation for Research and Treatment of Cancer Quality of Life Questionnaire-C30.

**Table 4:** Recommended Geriatric Assessment as stated in the ASCO Guideline for Geriatric Oncology [43].

Geriatric Assessment (GA) Domains	Tools/Measures
Function	Patient-reported IADL independence
Falls	Patient-reported falls
Comorbidity	Review medical history and medications
Cognition	Administered cognition tests: Mini-cog, BOMS, MMSE
Depression	GDS (questionnaire)
Nutrition	Patient-reported weight loss, MNA (administered)
If possible/applicable:	
Estimate Risk of Chemotherapy Toxicity	CARG or CRASH toxicity tool
Physical Performance	SPPB, TUG, gait speed (objective)

Abbreviations. IADL: instrumental activities of daily living, BOMC: Blessed Orientation-Memory-Concentration, MMSE: Mini-Mental State Examination, GDS: Geriatric Depression Scale, MNA: Mini Nutritional Assessment, CARG: Cancer and Aging Research Group, CRASH: Chemotherapy Risk Assessment Scale for High-Age Patients, SPPB: Short Physical Performance Battery, TUG: Timed Up and Go.
